# Anomalous Lehmann Rotation of Achiral Nematic Liquid Crystal Droplets Trapped under Linearly Polarized Optical Tweezers

**DOI:** 10.3390/molecules26144108

**Published:** 2021-07-06

**Authors:** Jarinee Kiang-ia, Rahut Taeudomkul, Pongthep Prajongtat, Padetha Tin, Apichart Pattanaporkratana, Nattaporn Chattham

**Affiliations:** 1 Department of Physics, Faculty of Science, Kasetsart University, Bangkok 10900, Thailand; jarinee.k@ku.th (J.K.-i.); 20032@students.isb.ac.th (R.T.); fsciacp@ku.ac.th (A.P.); 2 Department of Science, International School Bangkok, Nonthaburi 11120, Thailand; 3 Department of Materials Science, Faculty of Science, Kasetsart University, Bangkok 10900, Thailand; fscipop@ku.ac.th; 4 NASA Glenn Research Center, Cleveland, OH 44135, USA; padetha.tin-1@nasa.gov

**Keywords:** Lehmann effect, Lehmann rotation, liquid crystals, optical tweezers, achiral nematic

## Abstract

Continuous rotation of a cholesteric droplet under the heat gradient was observed by Lehmann in 1900. This phenomenon, the so-called Lehmann effect, consists of unidirectional rotation around the heat flux axis. We investigate this gradient heat effect using infrared laser optical tweezers. By applying single trap linearly polarized optical tweezers onto a radial achiral nematic liquid crystal droplet, trapping of the droplet was performed. However, under a linearly polarized optical trap, instead of stable trapping of the droplet with slightly deformed molecular directors along with a radial hedgehog defect, anomalous continuous rotation of the droplet was observed. Under low power laser trapping, the droplet appeared to rotate clockwise. By continuously increasing the laser power, a stable trap was observed, followed by reverse directional rotation in a higher intensity laser trap. Optical levitation of the droplet in the laser beam caused the heat gradient, and a breaking of the symmetry of the achiral nematic droplet. These two effects together led to the rotation of the droplet under linearly polarized laser trapping, with the sense of rotation depending on laser power.

## 1. Introduction

Chiral nematic liquid crystal (NLC), composed of rod-like molecules with symmetry breaking caused by the handedness of the molecules, can form a helical structure with molecular directors twisted around the axis in the chiral nematic phase. Droplets can be created from this type of structure and were found to be rotatable under unidirectional heat flux. This mechanism, so called Lehmann rotation, was first observed by Lehmann in 1900 [1] and later explained by Leslie [2]. The Lehmann effect under a temperature gradient was reproduced again after 100 years in an experiment with small drops of cholesteric phase suspended in an isotropic liquid [3,4]. Recently, there have been reports of Lehmann rotation in achiral molecules [5], which had been suggested theoretically by Brand et al. [6]. This discovery opens the door for liquid crystal scientists to search for more possibilities of finding the Lehmann effect in other macroscopic structures of achiral molecules. Other than subjecting to temperature gradient, there have been a few reports on the Lehmann effect caused by other initiators, e.g., electric field [7] and fluid flow [8,9]. In our experiment, we used linearly polarized light by means of optical tweezers to trap NLC droplets, which apparently caused unexpected rotation of the droplets.

Optical tweezers, discovered in 1970 by Ashkin [10], use a tightly focused laser beam to trap and move micron sized particles. It has become a powerful tool with extensive applications in biology, physical chemistry, and soft condensed matter physics [11,12,13]. Other than trapping and moving particles, stable levitation of a few micron sized spheres in air and in a vacuum was also demonstrated by Ashkin and Dziedzic [14,15]. The balancing of forces from the radiation pressure of the laser and from gravity forms a stable trapping potential [15]. Optical levitation is useful in many optomechanical systems [16], especially in ultrasensitive force detection [17]. In this experiment, we used linearly polarized optical tweezers to create an asymmetric macroscopic structure in achiral nematic droplets [18,19] and to optically levitate the droplets. Heat flux is generated by optical levitation whose strength and direction can be controlled by variation of laser power. Here we show that it is possible to exhibit Lehmann rotation of achiral nematic droplets subjected to heat flux under linearly polarized optical tweezers.

## 2. Materials and Methods

Nematic liquid crystal droplets were prepared by constant stirring of a mixture of 4′-n-pentyl-4-cyanobiphenyl (5CB, Sigma-Aldrich, St. Louis, MO, USA) and a cationic surfactant hexadecyltrimethylammonium bromide (CTAB, Sigma-Aldrich) in heavy water (D_2_O). NLC droplets suspended in heavy water were then inserted into a 100 μm thick glass cell for the optical tweezers experiment. The sample cells were investigated under crossed polarizers on an inverted microscope (Nikon Eclipse Ti) equipped with an optical tweezers setup.

**Figure 1:**
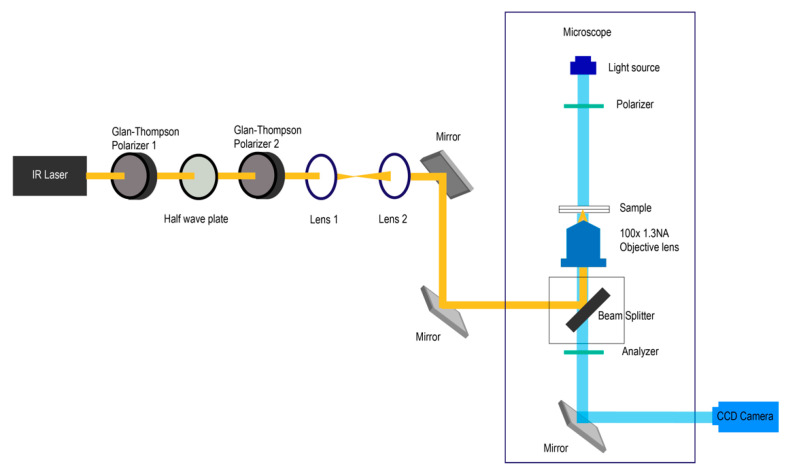
Schematic illustration of the optical tweezers setup on an inverted microscope.

This is shown in Figure 1. A 1064 nm Ytterbium-doped fiber laser was aligned through a Glan-Thompson polarizer for complete linear polarization of the beam. A half-wave plate was inserted for rotation of the polarization plane with the second Glan-Thompson polarizer in the beam path to ensure a correct plane of required polarization. The beam was collimated through a beam expander composed of two lenses to fill the back aperture of an objective lens (Nikon Plan Fluor 100× 1.3 NA) of the microscope. Observations were made and recorded with the CCD camera through the same objective lens. Two different CCD cameras, Basler acA1920-150uc and Sony Handycam HDR-SR12E, were used for different purposes. Basler CCD camera directly captured an infinite distance image from the parallel beam created by an objective lens onto its CCD chip without any optics in front of the camera. Images of higher resolution were taken by this camera. The second CCD camera, Sony Handycam HDR-SR12E, was connected to the camera port of a microscope via a C-mount. This connection had lenses in front of the CCD chip enabling an auto-focus function of this CCD camera, because the droplet moved out of focus of the objective lens in the experiment due to laser radiation pressure, and refocusing the droplet into view by moving the objective lens was not possible since the objective lens was used for both laser tweezers purpose and viewing purpose. If we move the objective lens, a laser focused point will be altered, resulting in the droplet position changing with respect to the focus of a laser. The second CCD camera gave lower image resolution but was suitable for the analysis of droplet motion.

## 3. Results and Discussion

**Figure 2:**
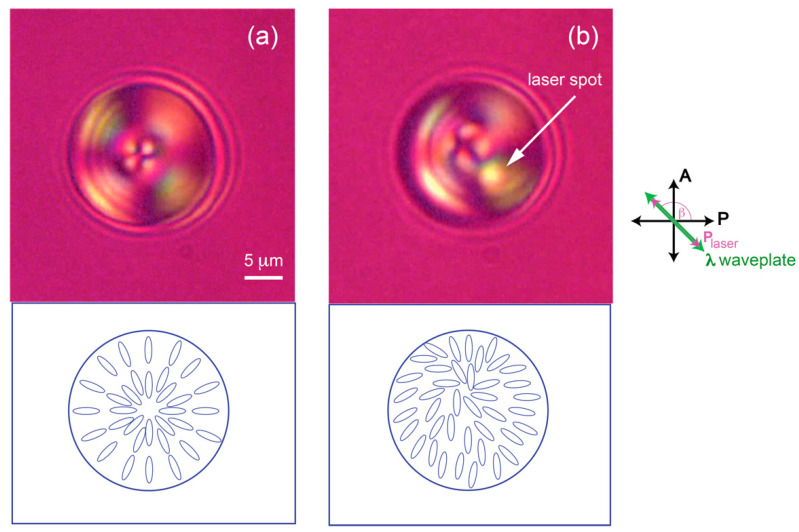
Microscopic images taken under crossed polarizers and a one-wavelength plate inserted diagonally for director orientation mapping. (**a**) A 19-μm radial NLC droplet suspended in water. The droplet configuration is spherically symmetric, the so-called ‘radial hedgehog’. (**b**) By applying the linearly polarized laser onto the droplet, the defect in the middle shifted slightly to the side along the polarization direction of the laser, β = 135° with respect to a horizontal direction. This distorted configuration caused by the alignment of the molecules in the laser beam pointed toward the laser polarization direction, thus pushing the defect to the side.

Symmetric radial NLC droplets, the so-called radial hedgehog, were observed with no laser applied as shown in Figure 2a. During laser trapping experiment, a radial hedgehog defect in the middle of the NLC droplets was pushed off center slightly depending on laser power. Figure 2b illustrates the off-centered defect in an optical trap after increasing the laser power to 298 mW with its polarization set at an angle β = 135° with respect to the horizontal axis. Molecules inside the droplet tried to orient themselves along the direction of laser polarization due to the optical torque $\langle \left(\;\vec{n}\cdot \vec{E}\right)\left(\vec{n}\times \vec{E}\right)\rangle$ on the molecules, where $\vec{n}$ is the molecular director and $\vec{E}$ is the electric field of the laser along the polarization direction. Symmetry breaking occurred in a radial configuration resulting in the defect being pushed off center [18,19] as shown in Figure 2b. As previously reported by Brasselet [18] and Phanphak [19] for linearly polarized optical tweezers, radial NLC droplets were stably trapped with no rotation observed. Mapping of director configuration was obtained by inserting a one-wavelength plate diagonally at an angle of 135° to the horizontal axis. Mapping of director orientation with birefringence color under crossed polarizers and a one-wavelength plate inserted can be modeled as in ref. [20].

**Figure 3:**
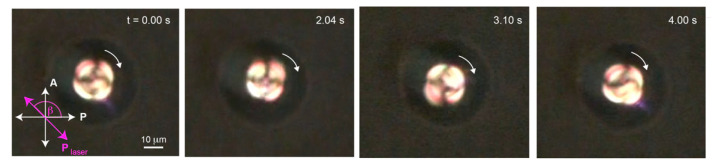
An NLC droplet under linearly polarized laser trap was found rotating clockwise at laser power 298 mW. The laser polarization direction was set at an angle β = 135° with respect to the horizontal (Supplementary video available).

**Figure 4:**
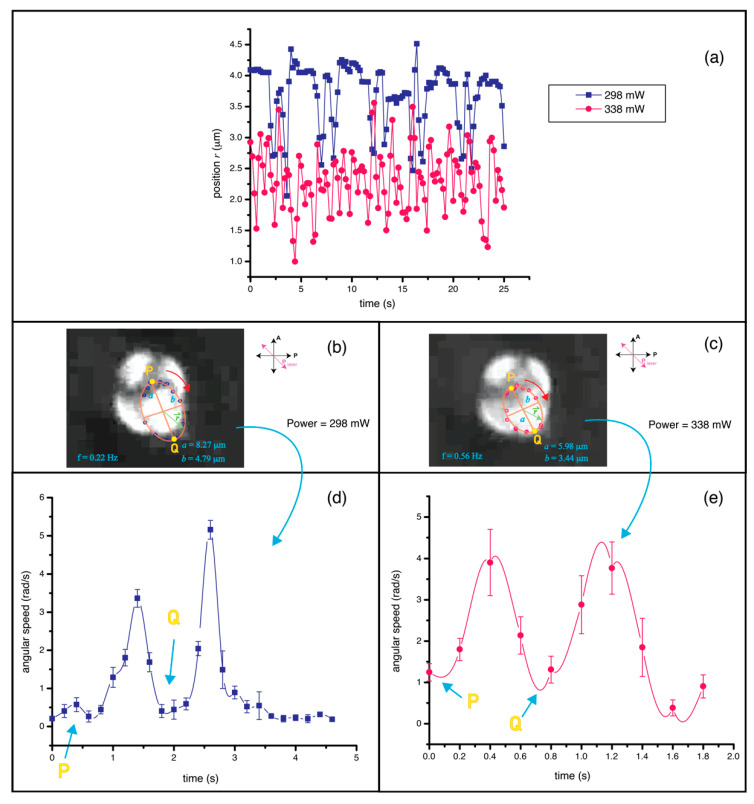
(**a**) Plot of the radial hedgehog defect position of 19-μm NLC droplet. The position *r* was measured with respect to the center of the elliptical trajectory (shown in (**b**,**c**)), during clockwise rotation at 298 mW and 338 mW. The period of rotation was 4.54 s and 1.79 s for 298 mW and 338 mW, respectively. (**b**,**c**) Tracking of the elliptical trajectory of the defect showing non-uniform angular speed. (**d**,**e**) Angular speed of the defect at 298 mW and 338 mW, respectively. The defect rotation was very slow at points P and Q along the direction of laser polarization for 298 mW laser trapping. The effect is less severe for higher laser power.

However, in our experiment, a stable trap was only observed in linearly polarized laser trapping whilst slowly increasing the laser power from 124 mW to 298 mW. After a short moment after reaching 298 mW, the defect started rotating around the laser spot in a clockwise direction as shown in Figure 3. We analyzed the motion by tracking the defect using Tracker video analysis from Open Source Physics (OSP) depicted in Figure 4a. The experiment was conducted for various sizes of droplets ranging from 14 μm to 48 μm. A 19-μm droplet was selected for detailed investigation.

Figure 4a shows the position of defect over time in clockwise rotation at laser powers of 298 mW and 338 mW. The position *r* of the defect was measured with reference to the center of the elliptical trajectory depicted in Figure 4b. The ellipse appeared to be smaller with increasing laser power, as can be seen in Figure 4c for the power of 338 mW. During the defect rotation, the motion was non-uniform. The blue square plot in Figure 4a shows that, for one period of rotation, T = 4.54 s, the defect spends more time at point P than at any other points. The motion was slowed down again at point Q in the trajectory. This non-uniformness was less severe at higher power as illustrated in the pink plot of Figure 4a for the power of 338 mW. We analyzed the angular speed of the defect for both laser powers (Figure 4d,e) and found that the contrast of speed was quite high for the lower laser power. The defect preferred to stay at the point along the polarization direction of the laser (point P and Q) as it fell into the linear polarization trapping potential, and speeded up at other positions along the long axis of the ellipse.

**Figure 5:**
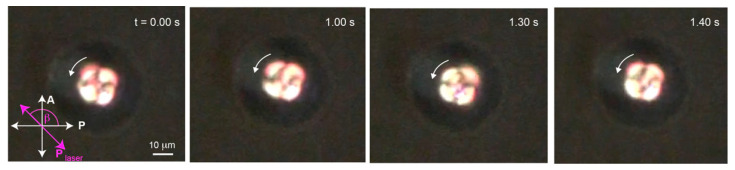
The same 19-μm NLC droplet as in Figure 3 rotated in the reverse direction under the same linearly polarized laser trap at a higher laser power of 635 mW (Supplementary video available).

When the laser power was increased further than 338 mW, the rotation stopped, and started again in a reverse direction at a higher threshold power. For this 19-μm droplet, the threshold power for rotating in a counterclockwise direction, shown in Figure 5, is 635 mW. It also rotated in an elliptical trajectory with semi-major axis length *a* = 1.50 μm and semi-minor axis length *b* = 0.45 μm with a higher frequency of rotation of 0.714 Hz. The elliptical shape was much smaller than with a lower power clockwise rotation. When the laser power reached 830 mW, the droplet stopped rotating and was pushed out of the laser trap at 1.90 W.

Previous observation was done when laser polarization made an angle of β = 135° to the polarizer. Different angles of β at 0°, 45°, and 90° were tried, to check our setup. Changing the angle of polarization changed the elliptical trajectory. The long axis of the elliptical path, as in Figure 4b,c, followed the axis of laser polarization change. Clockwise rotation was still observed at low power and counterclockwise rotation was observed at high power.

**Figure 6:**
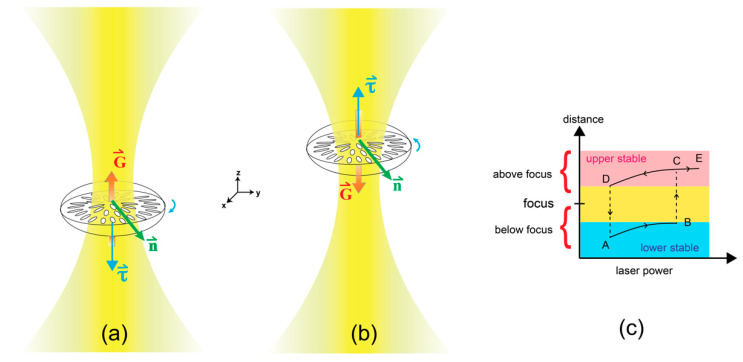
Schematic illustration of the droplet position at (**a**) low power and (**b**) higher power of a laser when slowly increasing the power of the trapping laser. $\vec{G}$ represents direction of temperature gradient due to laser intensity. The temperature gradient points toward the laser focus. $\vec{n}$ is the direction of average molecular directors of the distorted radial droplet. $\vec{\tau }$ is torque direction of the droplet from $\vec{\tau }=\nu \vec{n}\times \left(\vec{n}\times \vec{G}\right)$, which resulted in clockwise rotation in (**a**) and counterclockwise rotation in (**b**). (**c**) Particle position with laser power illustrated roughly the observation of Ashkin’s experiment in optical levitation [15].

This anomalous rotation in a clockwise and counterclockwise direction can be explained by the fact that along the laser trapping axis there is the gradient temperature, $\vec{G}$, pointing toward the focus of the laser. The location of the droplet in the laser trap can be determined from the competition of gravitational force and the radiation pressure of the laser. The beam waist of the focused laser through 100× objective lens was approximately 1 μm which is smaller than the particle size; therefore, this optical tweezers experiment can be considered using a ray optics regime. Optical levitation of a particle in a laser trap, with power, can be explained by the hysteresis loop, in Ashkin’s experiment [15] on a 19-μm solid glass sphere with TEM00 mode laser, depicted in Figure 6c. The hysteresis plotted is for a case in which the beam waist is smaller than particle size, which is the same as in our case. When the particle is lifted to the focal point, the vertical force is at a minimum. Our experiment followed along the track from A to B and to C and E in the hysteresis loop by slowly increasing the laser power. From A to B in the region of lower stability or when below focus, the thermal gradient $\vec{G}$ pointed in +z direction. The Lehmann rotation torque according to Leslie [2] takes the form:

(1) $$\vec{\tau }=\nu \vec{n}\times \left(\vec{n}\times \vec{G}\right),$$

where ν is the Lehmann coefficient and $\vec{n}$ is the average nematic director. Torque points along −z direction in the lower stable region, which results in clockwise rotation of the droplet, is shown in Figure 6a. By slowly increasing the laser power, the rotation stopped in our experiment and restarted in a reverse direction at higher power as shown in Figure 5. We did not find an abrupt reversal of rotational direction from clockwise to counterclockwise direction, as the hysteresis loop suggested, from point B to C. When the droplet is trapped at the focal point, there is no temperature gradient, thus no rotation was observed. By continually increasing the laser power, the droplet was levitated to the region above the focal point, then reverse rotation appeared as depicted in Figure 6b. At this point, the droplet reached point C in the hysteresis loop. By keeping the power increased, the droplet continued to point E and was pushed off the optical trap.

Notice that the upper stable can also happen at low power laser if the trapping starts from high power and reduces to lower power. We observed this case experimentally; however, for detailed investigation, we only increased the laser power in a forward direction.

The NLC droplet itself belongs to D_∞_ point group symmetry which should not show any rotation due to the Lehmann effect [6]. However, Lehmann rotation of the structure composed of achiral molecules has been reported [5,9] and explained theoretically [6]. In our experiment the droplet itself, although composed of achiral molecules, was distorted due to the laser polarization direction as shown in Figure 2b. The configuration could possess mirror symmetry after distortion with the mirror plane in the *x*-*y* plane, as shown in Figure 6a; however, the gradient of laser intensity along the *z*-axis destroys this mirror symmetry by orienting the molecules with stronger force near the laser focus. Therefore, a droplet trapped in the lower stable region or upper stable region is considered to possess C_1_ point group symmetry. According to Brand et al. [6], the C_1_ structure is enough to perform the Lehmann effect in a heat gradient.

**Figure 7:**
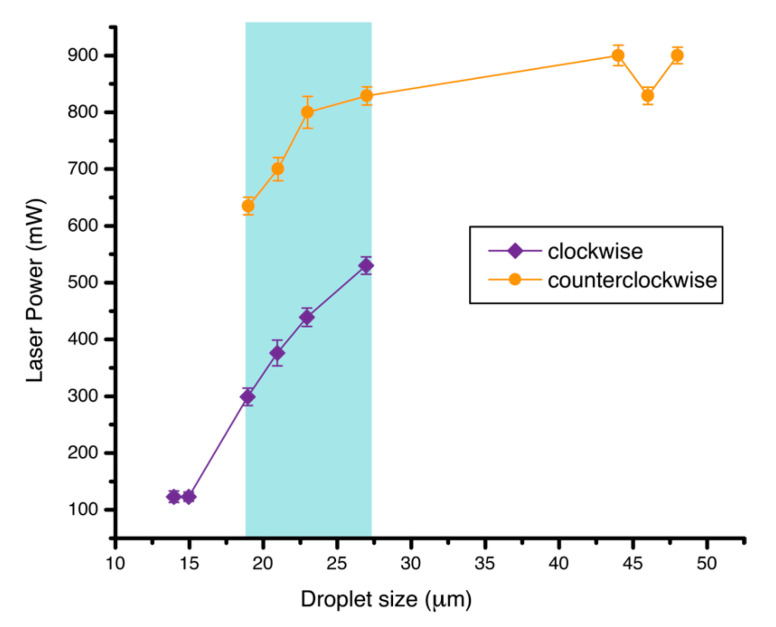
Plot of threshold laser power for droplet rotation in clockwise and counterclockwise directions with droplet size. Notice that there is no counterclockwise rotation of small droplets at high power since the droplets were too small, so they were pushed off the trap at high power. There is no clockwise rotation of larger droplets at low power because the droplets were too heavy for rotation. The force from a low power laser could be too small to initiate droplet motion.

The rotation mechanism was also investigated for droplets of other sizes. The plot in Figure 7 shows the threshold power of the laser for droplet rotation in a clockwise and counterclockwise direction, along with droplet size. The most suitable size of the droplet to observe both clockwise and counterclockwise rotation ranges from 19 to 27 μm. Below this range, the droplet is too small so that it only shows rotation in the lower stable region below the focus of laser. At higher laser power, it was pushed out of the optical trap. For the droplets larger than this range, the low power laser was not enough to drive the rotation, thus only counterclockwise direction occurred in the upper stable region.

**Figure 8:**
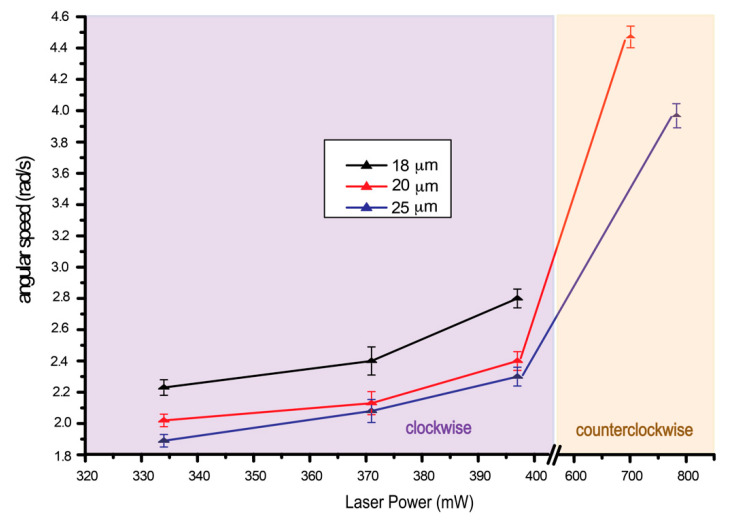
Plot of angular speed with laser power of a radial hedgehog defect under linearly polarized laser tweezers. Notice that we can only record one set of data for counterclockwise rotation for each droplet size since the power range for counterclockwise rotation is very small and by increasing the laser power higher than the last data plotted, the droplet was pushed out of the laser trap. For an 18 μm droplet, no counterclockwise rotation was observed, analogous to the observation in Figure 7.

Further analysis of angular speed for clockwise and counterclockwise rotation is depicted in Figure 8. It can clearly be concluded from the plot that angular speed of the droplet depends on droplet size and laser power. At low laser power, the droplet rotates clockwise with low angular speed, and at high laser power, the droplet rotates much faster in a counterclockwise direction.

In conclusion, we have shown that achiral nematic liquid crystal can form an asymmetric macroscopic structure under laser trapping, which is sufficient to generate Lehmann rotation under the heat gradient of a linearly polarized laser beam. The rotational sense of the droplet depends on heat gradient direction which can be determined by location of the droplet on the laser beam axis with respect to the laser focal spot. This mechanism, driven by heat flux of the laser, can also be applied to other macroscopically asymmetric structures. Bent core liquid crystals are suggested as a candidate for future investigation.

## Data Availability

The data presented in this study are available in Supplementary Material.

## Supplementary Materials

The Supplementary Materials are available online.

## Sample Availability

Samples of the compounds are not available from the authors.
